# TRF2 is recruited to the pre-initiation complex as a testis-specific subunit of TFIIA/ALF to promote haploid cell gene expression

**DOI:** 10.1038/srep32069

**Published:** 2016-08-31

**Authors:** Igor Martianov, Amandine Velt, Guillaume Davidson, Mohamed-Amin Choukrallah, Irwin Davidson

**Affiliations:** 1 Department of Functional Genomics and Cancer, Institut de Génétique et de Biologie Moléculaire et Cellulaire, CNRS/INSERM/UDS. 1 Rue Laurent Fries, 67404, Illkirch Cédex France; 2 The Friedrich Miescher Institute, Maulbeerstrasse 66, 4058, Basel, Switzerland

## Abstract

Mammalian genomes encode two genes related to the TATA-box binding protein (TBP), TBP-related factors 2 and 3 (TRF2 and TRF3). Male *Trf2*^−/−^ mice are sterile and characterized by arrested spermatogenesis at the transition from late haploid spermatids to early elongating spermatids. Despite this characterization, the molecular function of murine Trf2 remains poorly characterized and no direct evidence exists to show that it acts as a *bona fide* chromatin-bound transcription factor. We show here that Trf2 forms a stable complex with TFIIA or the testis expressed paralogue ALF chaperoned in the cytoplasm by heat shock proteins. We demonstrate for the first time that Trf2 is recruited to active haploid cell promoters together with Tbp, Taf7l and RNA polymerase II. RNA-seq analysis identifies a set of genes activated in haploid spermatids during the first wave of spermatogenesis whose expression is down-regulated by Trf2 inactivation. We therefore propose that Trf2 is recruited to the preinitiation complex as a testis-specific subunit of TFIIA/ALF that cooperates with Tbp and Taf7l to promote haploid cell gene expression.

Regulated initiation of transcription by RNA polymerase II (Pol II) involves formation of a preinitiation complex (PIC) over the transcription start site (TSS). In addition to RNA polymerase II (Pol II), the PIC comprises a set of general transcription factors TFIIA, B, D, E, F, and H as well as the mediator complex and a set of DNA repair factors^1^,^2^,^3^,^4^. TFIID is itself a macromolecular complex composed of the TATA-binding protein (TBP) and a set of 13–14 evolutionary conserved TBP-associated factors (TAFs)^5^,^6^. Among the TFIID subunits, TBP has been extensively studied as a key factor for all three nuclear RNA polymerases (Pol I, Pol II and Pol III) leading to the idea that it is a ‘universal transcription factor’^7^. Nevertheless, its universal status was called into question by the discovery of several TBP related factors (TRFs): TRF1, TRF2 (also called TLF and TLP) and TRF3 (also called TBP2)^8^,^9^,^10^,^11^,^12^.

TBP is a bipartite protein with a variable N-terminal domain, but a highly conserved C-terminal domain shared with the TRFs and forming a saddle-like structure with a concave surface that binds the consensus TATA element DNA and a convex surface that interacts with TAFs as well as the TFIIA and TFIIB components of the PIC^13^,^14^,^15^,^16^. TRF1 was first described in Drosophila and shows 60% amino acid identity to the C-terminal domain of TBP. TRF1 binds the TATA-element together with TFIIA and TFIIB and can replace TBP in transcription of TATA-dependent promoters^17^,^18^. TRF3 (TBP2), found only in vertebrates also binds the TATA-element in association with TFIIA and TFIIB to activate transcription^19^,^20^,^21^,^22^. In contrast, TRF2 found in all metazoans, is the most divergent from TBP with only 40% identity in the C-terminal domain^8^,^12^. Like TRF1 and TRF3, TRF2 also binds TFIIA, but it does not selectively recognize the TATA element^23^,^24^,^25^,^26^.

The functions of TRF2 have been studied in different organisms. In *Drosophila*, TRF2 binds chromatin at different sites from TBP and ChIP-seq analyses revealed its binding at the DPE motif in a large group of TATA-less promoters^27^,^28^,^29^. TRF2 was shown to be essential for embryogenesis in Drosophila, Xenopus and Zebrafish^30^,^31^. In Xenopus and Zebrafish, genes whose expression is selectively dependent on either TBP or TRF2 principally during early development have been identified^12^. In contrast, mouse genetics showed that Trf2 is not required for embryogenesis and adult *Trf2*^−/−^ mice are morphologically indistinguishable from their wild-type (WT) littermates^32^,^33^. Nevertheless, male *Trf2*^−/−^ mice are sterile and characterized by an arrest of spermatogenesis at the transition from late haploid spermatids to early elongating spermatids. Chromatin organization of mutant haploid round spermatids is disorganized by fragmentation of the chromocenter, a structure formed by pericentric heterochromatin of all chromosomes^34^. As a result of this disorganization, apoptosis occurs when these cells initiate the elongation process.

Despite this characterization of its essential role in spermatogenesis, the molecular function of murine Trf2 remains poorly characterized. Analyses of *Trf2*^−/−^ testes suggested that several haploid-expressed genes were down-regulated^32^,^33^. Nevertheless, these analyses are complicated by the fact that many haploid transcripts are stored in elongating spermatids to be translated at later stages after the arrest of active transcription. With the loss of these elongating spermatids in the mutant animals, the levels of these transcripts are reduced, but direct evidence that Trf2 is actively involved in their transcription is lacking. We previously showed that Trf2 interacts with TFIIA and its testis expressed paralogue ALF (TFIIA-like factor), but that unlike many other transcription factors, Trf2 was readily extracted from the nucleus under low salt conditions^35^. Despite these observations, the presence of Trf2 in the chromatin-associated PIC at promoters in testis has not been demonstrated. There is therefore no direct evidence that murine Trf2 acts as a *bona fide* transcription factor and that the effects seen upon its ablation are due to altered gene expression.

We show here that Trf2 forms a complex with TFIIA and ALF that is chaperoned in the cytoplasm by several heat shock proteins. We also demonstrate for the first time that Trf2 is recruited to active haploid cell promoters, not as a substitute for Tbp, but together with Tbp, Taf7l and Pol II. RNA-seq analysis identifies a set of testis genes activated around post-natal day 21 when the haploid spermatids are formed in the first wave of spermatogenesis and whose expression is down-regulated by Trf2 inactivation. We therefore propose that Trf2 is recruited to the PIC together with Tbp and Taf7l as a testis-specific subunit of TFIIA/ALF to promote haploid cell gene expression.

## Results

### Addition of a C-terminal tag on endogenous Trf2

**Figure 1:**
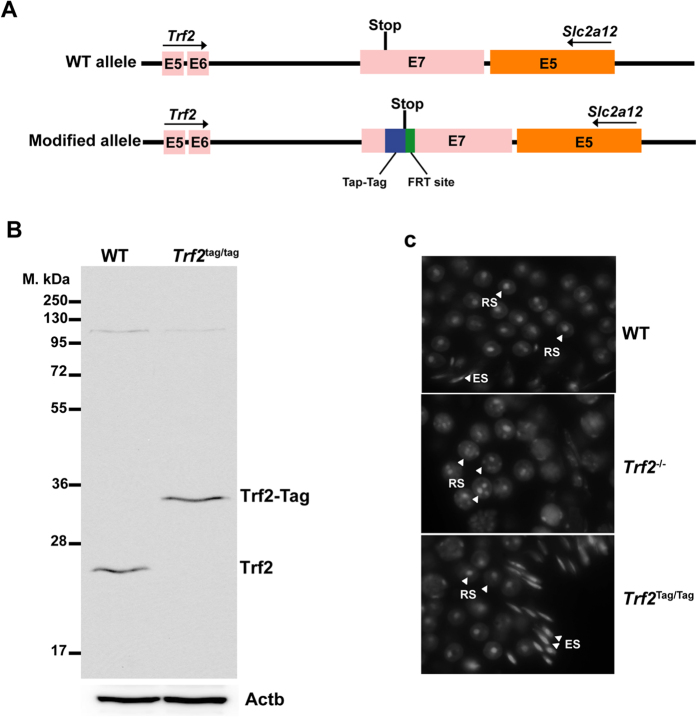
Generation of *Trf2*^tag/tag^ mice. (**A**) Schematic representation of WT and modified *Trf2* alleles with those of the partially overlapping *Slc2a12* gene. Exons (E) are indicated along with the Trf2 stop codon, the location of the Tap-Tag and the FRT sites. (**B**) Immunoblot on protein extracts from testis of WT and *Trf2*^tag/tag^ testes. Molecular mass markers are indicated on the left of the panel. (**C**) Hoechst staining of histological sections from mice of the indicated genotypes. RS shows round spermatids with a prominent chromocenter, PS shows pachytene spermatocytes and ES elongate spermatids.

To better characterise the molecular function of Trf2 and to overcome the lack of reliable ChIP-grade and immunoprecipitating antibodies, we used homologous recombination to generate ES cells and mice expressing endogenous Trf2 tagged on its C-terminus with a 6xHis-Tev-3xFlag tag (Fig. 1A). This Tap-tag was designed to facilitate both protein purification and ChIP. Western blot analysis showed that homozygous *Trf2*^tag/tag^ mice expressed the Trf2-Tag protein in their testes at levels comparable to WT littermates (Fig. 1B). *Trf2*^tag/tag^ mice were born at Mendelian ratio and did not display any obvious abnormalities (data not shown). More importantly, *Trf2*^tag/tag^ male mice were fertile indicating that Trf2-tag was functional and supported spermatogenesis. This can be seen in histology sections from testis showing that haploid round spermatids from WT and *Trf2*^tag/tag^ mice contained an intact chromocenter, whereas those from *Trf2*^−/−^ mice comprised a fragmented chromocenter (Fig. 1C). In addition, elongate spermatids were readily visible in sections from *Trf2*^tag/tag^ mice showing that spermatogenesis was not arrested in these animals. These observations altogether indicated that C-terminal tagged Trf2 was expressed and functional.

### Trf2 associates with TFIIA and ALF

**Figure 2:**
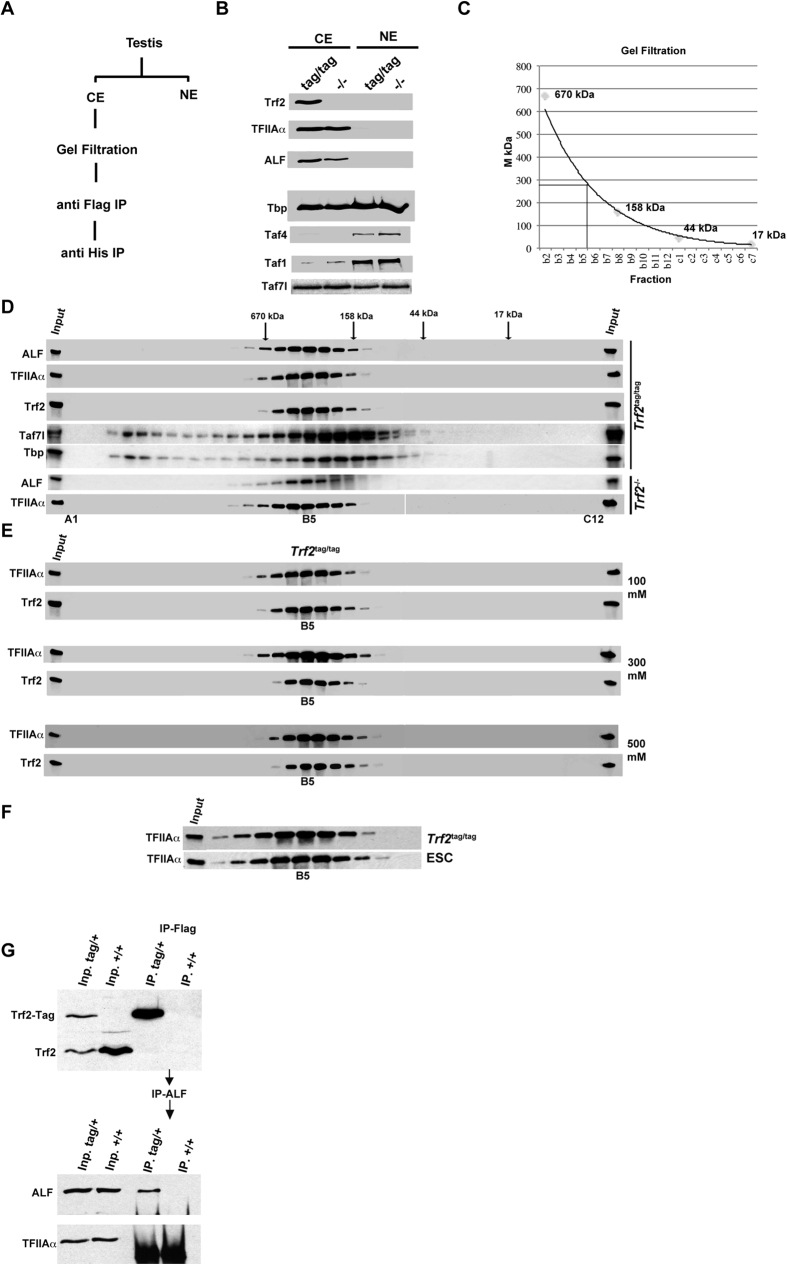
Trf2 is co-purified with TFIIA and ALF. (**A**) Schematic of purification of testis into cytoplasmic (CE) and nuclear extract (NE) before gel filtration and tandem affinity purification. (**B**) Immunoblots of cytoplasmic and nuclear extracts from *Trf2*^tag/tag^ and *Trf2*^−/−^ testis. (**C**) Elution of molecular mass markers on the gel filtration column. (**D**) Immunoblots on fractions following gel filtration of cytoplasmic extracts from *Trf2*^tag/tag^ and *Trf2*^−/−^ testes. (**E**) Immunoblots on fractions following gel filtration performed at the indicated KCl concentrations. (**F**) Immunoblots on fractions following gel filtration of cytoplasmic extracts from *Trf2*^tag/tag^ testes and murine embryonic stem cells. (**G**) Upper panel shows immunoblots on cytoplasmic extracts (Input, Inp.) from WT and *Trf2*^+/tag^ heterozygous mice indicating the positions of the native and tagged proteins. The right hand lanes show the Flag immunoprecipitates from the corresponding Input fractions in the left lanes. The lower panel shows the immunoblots of the anti-ALF IP performed on the fractions eluted from the Flag IP in the upper panel.

We previously reported that Trf2 interacts with high affinity with TFIIA and its testis specific paralogue ALF^35^, but it is still not clear if other partners exist. To address this, we purified Trf2 from testis protein extracts of *Trf2*^tag/tag^ mice (Fig. 2A). After preparing nuclear and cytoplasmic protein extracts, we found as previously reported^23^,^35^ that the majority of Trf2 was found in the cytoplasmic fraction together with TFIIA and ALF (Fig. 2B). The efficiency of the differential extraction was monitored with TFIID subunits Taf1 and Taf4 that as expected were found almost exclusively in the nuclear extracts and Taf7l, that as previously described^35^,^36^, was found in both the cytoplasmic and nuclear fractions. Similarly, Tbp that is a subunit of several different complexes in addition to TFIID was found in both cytoplasmic and nuclear fractions. The fact that Trf2 and ALF were detected by immunostaining in the nucleus of round spermatids, but that they were found in the cytoplasmic fraction after extraction with low ionic strength buffer indicated their low affinity for chromatin^33^,^35^.

Cytoplasmic extracts were adjusted to 100 mM KCl and loaded on a gel filtration column. Western blot analysis of the eluted fractions showed that Trf2 elution centred around fraction B5 (corresponding to an apparent molecular mass of approximately 280 kDa) along with TFIIA and ALF (Fig. 2C,D). Importantly, no low molecular mass form corresponding to free Trf2 was observed indicating that the totality of Trf2 existed as a complex with TFIIA and/or ALF. This complex was stable as increasing the KCl concentration to 0,5 M did not result in dissociation of Trf2 from TFIIA and ALF (Fig. 2E).

Gel filtration of protein extracts from *Trf2*^−/−^ testes showed that TFIIA and ALF eluted around the same B5 fraction (Fig. 2D). The absence of Trf2 would result in a loss of only 30 kDa that would not be expected to change the mobility of the complex. However, this observation indicated that TFIIA and ALF form the 280 kDa complex in absence of Trf2 and as this complex eluted with the same apparent molecular mass in extracts from *Trf2*^tag/tag^ and *Trf2*^−/−^ testes, the absence of Trf2 did not result in the loss of an additional major subunit of this complex. Hence, TFIIA and ALF are the major interacting partners of Trf2 in testes as if other proteins interacted directly with Trf2, its loss would result in a more important change in the apparent molecular mass of the complex. This idea is further supported by the observation that TFIIA eluted in complex of a similar molecular mass from cytoplasmic extracts of mouse embryonic stem cells (ES) cells that do not express Trf2 (Fig. 2F). Thus, Trf2 and any interacting proteins did not contribute a majority of the mass of the TFIIA/ALF complexes.

Notwithstanding the above conclusion, the Trf2 complex eluted with a higher apparent molecular mass than expected by addition of the individual molecular masses of Trf2-Tag (30kDa) and TFIIA (66 kDa) or ALF (82 kDa). The higher molecular mass could be explained if the complex were a dimer formed through dimerization of Trf2 or the TFIIA/ALF components of the complex. To test this, we prepared extracts from heterozygous *Trf2*^tag/+^ and WT mouse testis and performed anti-Flag immunoprecipitation (IP). Trf2-tag, but not native Trf2 was present in the IP from the *Trf2*^tag/+^ extracts, and neither was present in the extracts from the WT testis (Fig. 2G) indicating that Trf2-tag did not co-precipitate native Trf2 and hence they did not dimerise. We then eluted the proteins from the IPs and reprecipitated them with ALF antibody. Under these conditions, ALFα could be immunoprecipitated, but not TFIIAα. Hence, Trf2 did not heterodimerise and the Trf2-ALF complex did not comprise TFIIAα showing there was no heterodimerisation via the subunits of TFIIA or ALF.

**Figure 3:**
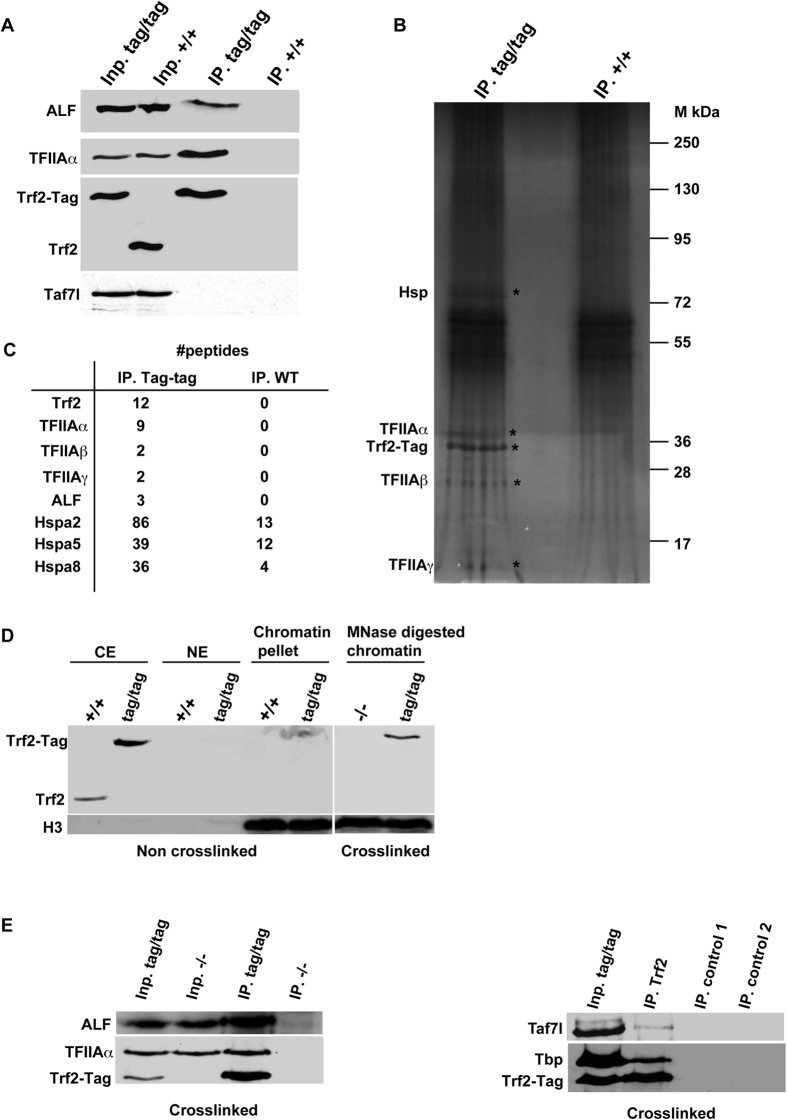
Composition of the cytoplasmic Trf2 complex. (**A**) Immunoblots of the fractions obtained by tandem affinity purification. (**B**) SDS-PAGE and silver nitrate staining of the immunoprecipiated fractions. The locations of tagged Trf2 and the TFIIA subunits are indicated. (**C**) The number of peptides obtained for each protein after mass-spectrometry analysis of the precipitated fractions. (**D**) Immunoblots of CE, NE and chromatin pellet from non-crosslinked testis and the MNase digested chromatin from formaldehyde crosslinked chromatin. (**E**) Input fractions and Flag IPs from formaldehyde crosslinked chromatin. Control 1 is no antibody IP on the chromatin, control 2 is antibody and sepharose beads, but no input chromatin.

One potential subunit of the Trf2-TFIIA/ALF complex is Taf7l that has been suggested to interact with Trf2^37^. Immunoblot showed that Taf7l did not co-elute with Trf2, but as a distinct peak offset by 4 fractions with the major Taf7l complex eluting as a complex with an apparent molecular mass of around 170 kDa (Fig. 2D). Moreover, Taf7l was eluted in second high molecular mass peak. In agreement with this, Taf7l was not detected in the tandem Flag-histidine purifications from the *Trf2*^tag/tag^ testis (Fig. 3A). Cytoplasmic Taf7l therefore does not form a complex with Trf2. Moreover, the cytoplasmic fraction of Tbp co-eluted with Taf7l and not with Trf2-TFIIA, in particular Tbp and Taf7l coeluted in a high molecular weight fraction that perhaps corresponds to a minor quantity of cytoplasmic TFIID (Fig. 2D).

The above data suggested the 280 kDa complex comprised additional unidentified proteins that interact with TFIIA/ALF independently of Trf2 to make up the missing mass. To identify other potential subunits, we performed tandem affinity purification of Trf2-containing complexes from the combined B3–B7 fractions from the *Trf2*^tag/tag^ and WT mice as control. Western blot analysis confirmed the presence of TFIIA and ALF only in the Trf2 immunoprecipitates from the *Trf2*^tag/tag^ mice (Fig. 3A). Immunopurified Trf2-tag and the TFIIA α, β and γ subunits were also visible by silver nitrate staining in the *Trf2*^tag/tag^ IP (Fig. 3B). Sections from the SDS-PAGE gel containing these proteins and the higher molecular mass fractions were systematically excised and analysed by mass spectrometry. Peptides for Trf2, TFIIA α, β and γ were specifically found in the corresponding bands from the *Trf2*^tag/tag^ IP (Fig. 3C). Peptides for ALF were also selectively detected in the *Trf2*^tag/tag^ IP. In addition to the expected subunits, many other proteins were detected, especially in the 45–65 kDa region, but they corresponded to contaminants and were not specific for the *Trf2*^tag/tag^ IP.

Nevertheless, silver nitrate staining revealed a protein in the region around 75 kDa selectively in the *Trf2*^tag/tag^ IP. This band was excised and analysed by mass spectrometry revealing that it comprised Hspa2, Hspa5, and Hspa8. All of these proteins were present at much higher levels in the *Trf2*^tag/tag^ IP (Fig. 3C). These data showed that the cytoplasmic Trf2-TFIIA/ALF complex is chaperoned, likely by at least two molecules of one or several of the above heat-shock proteins that account for the apparent molecular mass of the complex.

### Trf2 is recruited to active promoters as a subunit of TFIIA/ALF

The above results showed that Trf2 associates with TFIIA/ALF in a complex that readily dissociates from chromatin. Nevertheless, TFIIA is a *bona fide* PIC component suggesting that Trf2 may also be recruited to the PIC through its stable association with TFIIA/ALF. Trf2 was not detectable in the residual chromatin pellet after preparation of the cytoplasmic and soluble nuclear extracts, while histone H3 was clearly specific to the chromatin fraction (Fig. 3D). Nuclei were then prepared from formaldehyde cross-linked testes in the same manner. To release proteins from fixed nuclei, we used micrococcal nuclease (MNase) digestion. Western blot analysis showed that Trf2 was retained on chromatin by cross-linking as was histone H3 (Fig. 3D). We then used anti-Flag M2 resin to precipitate tagged Trf2 from MNase digested cross-linked chromatin and found that TFIIA and ALF were co-precipitated from the *Trf2*^tag/tag^ chromatin, but not from that of *Trf2*^−/−^ testis (Fig. 3E). These results showed that the Trf2-TFIIA/ALF complexes were recruited to chromatin and could be captured by cross-linking. Hence, Trf2 interacted with TFIIA/ALF not only in soluble protein extracts, but also when recruited to chromatin consistent with the idea that Trf2 is recruited as a subunit of TFIIA/ALF.

TFIIA is a general transcription factor recruited to promoters together with TFIID as a PIC component. If Trf2 is recruited to promoters as a subunit of TFIIA/ALF, subunits of TFIID should also then be co-precipitated with Trf2 from MNase digested cross-linked chromatin. Indeed, Tbp was readily detected co-precipitating with Trf2 together with Taf7l (Fig. 3E). Altogether, our results were consistent with the idea that Trf2 is a germ cell-specific subunit of TFIIA/ALF and recruited to the chromatin associated PIC.

**Figure 4:**
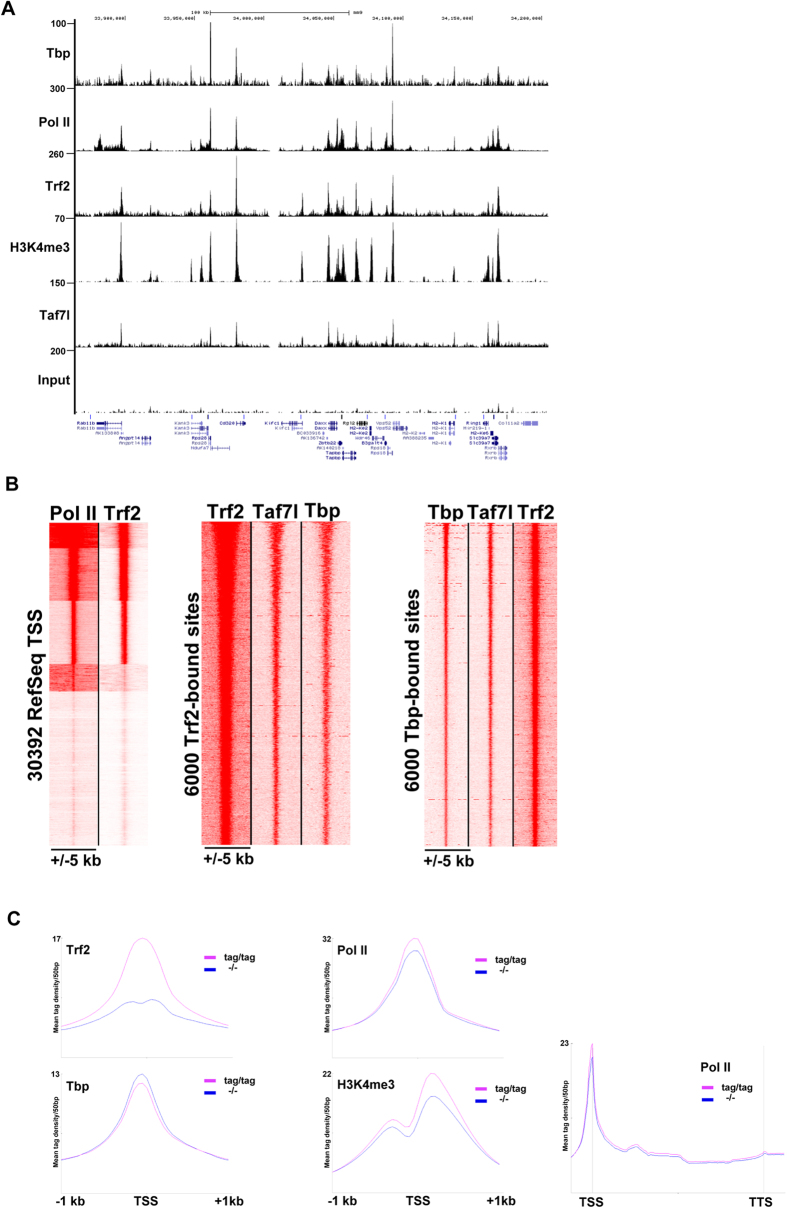
Trf2 occupies active promoters. (**A**) UCSC screen shot of ChIP-seq for the indicated factors over a 3-megabase region illustrating the colocalisation of Trf2, Tbp, Pol II, and Taf7l at active promoters. (**B**) Read density clustering showing, colocalisation of Trf2 with Pol II at a subset of promoters active in testis and co-localisation and proportionality of Trf2, Tbp and Taf7l occupancy at 6000 Trf2 bound sites and 6000 Tbp bound sites. (**C**) Meta-profiles of the ChIP-seq data for the indicated factors in *Trf2*^tag/tag^ and *Trf2*^−/−^ testis.

To test this idea, we performed ChIP-seq for Trf2, Pol II, Tbp and trimethylated lysine 4 of histone H3 (H3K4me3) to identify active promoters. ChIP was performed on chromatin from *Trf2*^tag/tag^ testes where for Trf2 we performed ChIP-reChIP on MNase digested chromatin using first anti-Flag antibody followed by an in house anti-Trf2 antibody. We performed this tandem ChIP-procedure as neither antibody individually gave an interpretable signal on either sonicated or MNase digested chromatin (see also below). Integration of these data sets showed that Trf2 bound to active H3K4me3-marked promoters along with Tbp, Pol II and Taf7l (Fig. 4A and see ref. 37). Around 12730 TSS were co-occupied by Trf2 and Pol II (Fig. 4B). Analysis of the 6000 most highly occupied Trf2 bound sites, showed not only co-localisation with both Tbp and Taf7l, but also a general correlation of binding of each factor at these sites (Fig. 4B). Sites, where Trf2 showed highest binding were also those showing highest Tbp and Taf7l occupancy. Similarly, analysis of the top 6000 TBP bound sites showed co-localisation with Trf2 and Taf7l (Fig. 4b).

We also performed ChIP-seq from *Trf2*^−/−^ testis chromatin. While the specific signal for Trf2 was lost in the chromatin from mutant testes, that of Tbp, Pol II and H3K4me3 was virtually unchanged (Fig. 4C). Similarly, Trf2 loss did not globally affect Pol II promoter occupancy and elongation. These data show that Trf2 is recruited to active TSS as part of the PIC, but that its loss did not globally affect PIC formation. We verified these data at the *Rpl37* and *Znf512b* promoters, that showed prominent Trf2 binding in ChIP-seq, by ChIP-qPCR after anti-Flag ChIP and tandem anti-Flag and anti-Trf2 ChIP. Flag-ChIP alone gave only poor enrichment of the promoter regions using chromatin from *Trf2*^tag/tag^ compared to chromatin from *Trf2*^−/−^ animals and to a negative intragenic region (Supplementary Fig. 1A). In contrast, tandem re-ChIP with the Trf2 antibody gave a more robust enrichment specific to the promoter regions and the chromatin from *Trf2*^tag/tag^ testis (Supplementary Fig. 1B). The ChIP-seq profiles for these two promoter regions showed a complete loss of the Trf2 signal in the chromatin from *Trf2*^−/−^ testis, whereas comparable Pol II signals were seen using chromatin from *Trf2*^tag/tag^ and *Trf2*^−/−^ animals (Supplementary Fig. 1C). Note also that the lower Trf2 signals at the TSS of the genes neighbouring *Znf512b* in the *Trf2*^tag/tag^ chromatin were also completely lost in the *Trf2*^−/−^, chromatin. These data showed the specificity of the Trf2 ChIP-seq protocol.

### Trf2 is required for normal haploid cell gene expression

**Figure 5:**
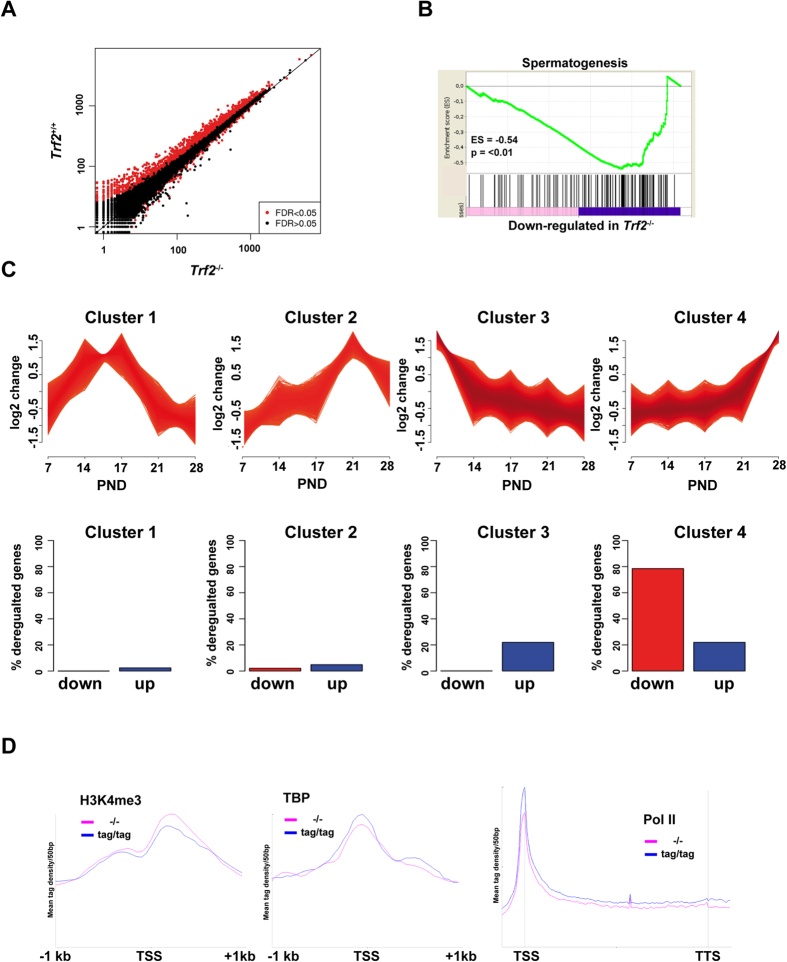
Trf2 promotes haploid cell gene expression. (**A**) Comparison of RNA-seq profiles from 21 day old testes from WT and *Trf2*^−/−^ mice. (**B**) Gene set enrichment analyses of the down-regulated genes in *Trf2*^−/−^ testes. (**C**) Gene expression data from public data set GSE39970 was organised into 4 clusters representing genes with different expression dynamics relative to the onset of the first wave of spermatogenesis between post-natal days (PND) 7 and 28. The lower part of the panel shows the % of the genes deregulated in the *Trf2*^−/−^ mice belonging to the different clusters. (**D**) Metaprofiles of the indicated factors at the promoters of the 711 down-regulated genes in WT and *Trf2*^−/−^ testis.

The above data showed that Trf2 was recruited to active promoters, but as its loss had no global effect on PIC formation and Pol II elongation its precise role in transcription was not clear. To address this, we performed RNA-seq from testes of WT and *Trf2*^−/−^ mice. As mentioned in the introduction, the loss of the elongate spermatid population in the mutant mice precluded a global analysis of testis RNA that would be strongly biased by the loss of the RNA-stored in this population. To circumvent this, we made RNA from 21 day-old testes corresponding to the appearance of round spermatids in the first wave of spermatogenesis, but before apoptosis of the round spermatids in the mutant animals. RNA-seq revealed 711 genes down-regulated in the mutant testis, but only 41 up-regulated (Fig. 5A). Amongst those down-regulated, 77 genes were down-regulated more than 4-fold and none more than 10-fold (Supplementary Table 1). Gene set enrichment analyses of down-regulated genes showed they were strongly associated with spermatogenesis (Fig. 5B).

The first wave of spermatogenesis occurs in a synchronised manner each germ cell type appearing at well-defined time points. At post-natal day (PND) 7 only spermatogonia and somatic cells are present. At PND14, the first pachytene spermatocytes appear. At PND20 the first round spermatids can be detected, and by PND30 spermatids have elongated. Each stage of spermatogenesis is characterised by activation and repression of a set of genes. Laiho *et al*.^38^ performed a comprehensive study of spermatogenesis gene expression at these different stages. We used data from PND7, 14, 17, 21 and 28 (GSE39970) to assign testis-expressed genes according to their expression patterns in 4 clusters (Fig. 5C). Genes in cluster 1 were expressed in spermatogonia and somatic cells and down-regulated at the onset of spermatogenesis. Clusters 2–3 represent genes highly up-regulated in pachytene spermatocytes and at the beginning of and during meiosis. The expression of most of these genes was down-regulated at later times in round spermatids. Genes in cluster 4 were up-regulated in haploid round spermatids beginning around PND20. We next assigned the genes deregulated by Trf2 loss to each cluster. Strikingly, around 80% of these genes belonged to cluster 4 showing that Trf2 loss mainly affected expression of genes that were activated or up-regulated in round spermatids (Fig. 5C).

As it has been suggested that Trf2 and Taf7l cooperate to regulate spermatogenesis^37^, we compared gene expression changes seen here with the public data for the *Taf7l*^−/y^ mice. However, only 15 genes were commonly down-regulated in both lines (Supplementary Table 1). Similarly, transcription factor CREMτ is an important regulator of haploid cell gene expression that occupies a large number of promoters and whose loss leads to early round spermatids apoptosis^39^,^40^. Nevertheless, of the promoters of the genes found deregulated here only 46 were occupied by CREM and only 14 genes were commonly down-regulated by CREM and Trf2 inactivation (Supplementary Table 1).

To assess the underlying mechanism of changes in gene expression upon Trf2 inactivation, we examined the recruitment of Tbp, and Pol II and H3K4me3 specifically at these genes in WT or *Trf2*^−/−^ testis. H3K4me3 levels were not reduced at these promoters, while Tbp levels were only mildly reduced in absence of Trf2 (Fig. 5D). The most noticeable difference was seen for Pol II where the levels of promoter paused and elongating Pol II were visibly reduced at these genes, contrary to the overall profile at all TSS where little change was seen. Nevertheless, the decrease was modest reflecting the modest changes in expression.

Altogether these data showed that Trf2 was recruited to the promoters of active genes to promote optimal recruitment of Pol II and expression of a set of haploid cell-expressed genes involved in spermatogenesis.

## Discussion

In this study, we show that Trf2 formed a stable complex with TFIIA/ALF. In absence of crosslink, the Trf2-TFIIA/ALF complexes are released from the nucleus even under low ionic strength conditions and are then chaperoned in the cytoplasmic extract by heat shock proteins. Consequently, little or no Trf2-TFIIA/ALF was found in the nucleus associated with chromatin and neither Tbp nor Taf7l co-eluted or co-precipitated with the cytoplasmic Trf2-TFIIA/ALF complexes. In presence of formaldehyde however, Trf2 was cross-linked to chromatin and co-precipitated with Tbp and Taf7l. We previously reported using immunostaining on testis that both Trf2 and ALF localise almost exclusively to the nucleus of late pachytene spermatocytes and haploid round spermatids^33^,^34^,^35^. Thus, as previously proposed^35^, in cells the Trf2-TFIIA/ALF complexes are nuclear, but are not tightly associated with chromatin and are released during the extraction procedure and then chaperoned in the cytoplasmic extract by heat shock proteins.

**Figure 6:**
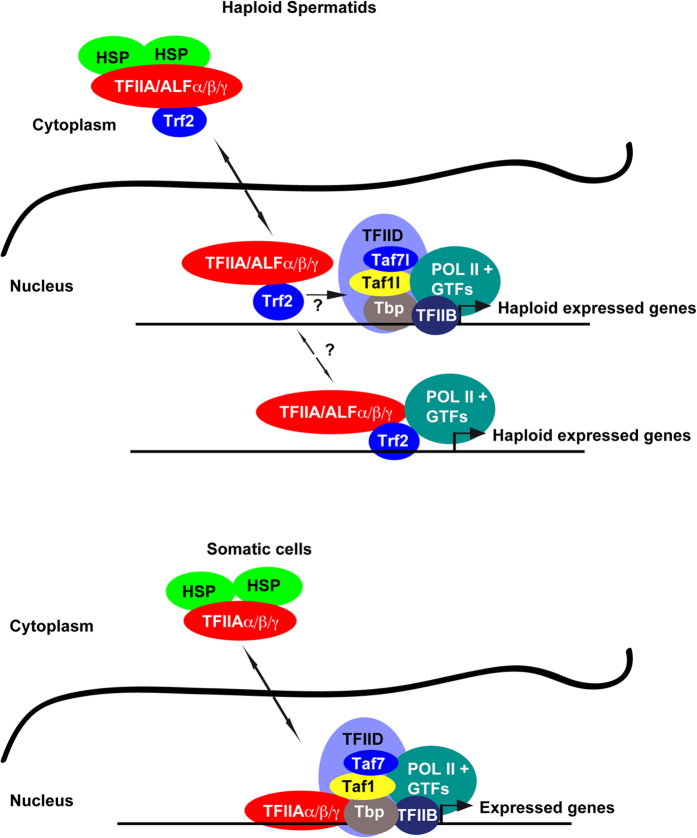
Presence of Trf2 alters PIC organisation. The figure shows a schematic summarising the results of this study. The Trf2-TFIIA/ALF complexes are weakly associated with the chromatin and readily extracted from the nucleus. They are chaperoned in the cytoplasm by heat shock factors. In haploid cells the PIC minimally comprises Tbp, Taf7l and Taf1l together with Pol II and the other general transcription factors (GTFs). As Trf2 is a subunit of TFIIA in haploid cells, Tbp cannot engage TFIIA in the canonical interaction seen in somatic cells. In contrast, in somatic cells in absence of Trf2, Tbp can interact with TFIIA. The presence of Trf2 as a subunit of TFIIA therefore modifies the organisation of the PIC in haploid cells.

Consistent with their presence in the nuclei of intact cells, ChIP-seq experiments revealed that Trf2 could be found associated with active promoters along with Pol II, Tbp and Taf7l. Importantly, the observation that Trf2 co-precipitated with Tbp and Taf7l from cross-linked chromatin indicated that Trf2 was bound to the promoters along with Tbp, and not in place of Tbp. This precludes the possibility that the ChIP-seq signal resulted from two mutually exclusive states, one where Trf2 was present in the PIC in absence of Tbp/TFIID and another where Tbp/TFIID was present in absence of Trf2. While these states may exist, the co-precipitation of these factors showed that at least at some stage all components were simultaneously bound to the promoters. Similarly, the sites strongest bound by Trf2 were also those that were strongest bound by Tbp and Taf7l, suggesting that occupancy reflects the transcriptional activity of the promoters and not selective and differential occupancy of specific classes of promoter by Tbp and Trf2. Although we cannot formally exclude the possibility that haploid gene expression is driven by two distinct PICs, one comprising Trf2-TFIIA/ALF, the other Tbp-Taf7l (Fig. 6), we favour a model in which recruitment of Trf2 along with Tbp-Taf7l is essential for optimal expression of haploid genes.

The finding that the Trf2-TFIIA/ALF complex was recruited to the PIC with Tbp and Taf7l does not imply direct interactions between these factors and raises the question of how Trf2 was recruited to the PIC. A common interface on Trf2 and Tbp is required for interactions with TFIIA and the Trf2-TFIIA interaction is much stronger than with Tbp such that Tbp and Trf2 cannot simultaneously interact with the same TFIIA molecule^26^,^23^. These observations suggest that the Trf2-TFIIA/ALF complex is not recruited to the PIC via Tbp-TFIIA interactions, but by an alternative pathway.

Zhou *et al*., reported that Taf7l interacts with Trf2^37^. However, Taf7l did not co-elute with Trf2-TFIIA and we did not detect Taf7l-Trf2 interactions by immunoblot or by mass spectrometry under non-crosslinked conditions. Alternatively, TFIIA interacts with the Taf4 subunit of TFIID suggesting that this interaction may recruit Trf2-ALF/TFIIA to the PIC^41^,^42^. Although Taf4 levels were strongly reduced in haploid cells^36^, this does not exclude the possible presence of Taf4 in the PIC. For example, strongly reduced levels of several TFIID subunits are observed in differentiating C2C12 myoblasts, but the residual levels form a TFIID complex that is recruited to promoters to drive myotube gene expression^43^,^44^. Also, recent electron microscopy studies of reconstituted PICs have revealed contacts between TFIIA and the small subunit of TFIIF that may contribute to recruitment of the Trf2-TFIIA/ALF complex^15^,^45^. Nevertheless, correct positioning of TFIIA to make these contacts may require the canonical Tbp-TFIIA interaction.

Interactions with DNA may also contribute to Trf2 recruitment to the PIC. Trf2 does not selectively recognise the canonical TATA element due to mutations in critical phenylalanine residues of its DNA binding surface^8^,^26^, however it may be able to recognise other features of promoter DNA, such as has been reported for *Drosophila* Trf2 that was enriched at ribosomal promoters with the polypyrimidine TCT initiator^46^. Given that we found murine Trf2 at all active promoters, its contacts if any with the DNA are likely to be in a non-specific rather than specific recognition of a given sequence.

While interactions of Trf2 with DNA or other PIC components may contribute to its recruitment, TFIIA or more specifically the Taspase I cleaved form of TFIIA^35^,^47^,^48^ is required for spermatogenesis. Oyama *et al*. reported that Taspase I inactivation or expression of a non-cleavable form of TFIIAα/β resulted in defective spermatogenesis and reduced Trf2 recruitment to transition protein and protamine promoters^49^. Oyama worked from the postulate that Trf2 was recruited only to promoters of spermatogenesis genes, whereas we found Trf2 associated with all expressed genes including house-keeping genes. Oyama *et al*. did not analyse the effect of TFIIAα cleavage on Tbp recruitment and more recently it has been shown that TFIIAα cleavage affects Tbp/TFIID regulation^50^. As both Trf2 and Tbp were present at the promoters of haploid cell genes, the non-cleavage of TFIIAα could therefore affect transcription by via the activity of Trf2 and/or Tbp.

Notwithstanding the interactions involved, our results suggest the conformation of the PIC at promoters in haploid cells differs from that in cells where Trf2 is absent. In absence of Trf2, Tbp can interact with TFIIA in the PIC, but in testis, Trf2 is an integral subunit of TFIIA such that the Tbp in the PIC cannot engage this canonical interaction seen in somatic cells (Fig. 6). Indeed, previous experiments have shown that Trf2 acts to repress basal transcription by such a mechanism^26^. In contrast, our data show that Trf2 promotes gene expression in haploid cells *in vivo* as its loss leads predominantly to down-regulated gene expression. RNA-seq identified 711 genes showing reduced expression when the haploid gene expression programme is activated in 21day old testes. While the majority of these genes are associated with spermatogenesis and/or selectively expressed in haploid cells, others such as *Stat4, Vav2* or *Aurkc* are much more widely expressed. Hence Trf2 contributes not only to expression of spermatogenesis genes, but more generally to haploid cell gene expression. On the other hand, we did not detect diminished expression of the protamine and transition protein genes whose expression, is low at this stage and increases only at later times and is known to be strongly diminished in *Trf2*^−/−^ testis^32^. It is therefore likely that analysis at later stages of haploid cell development, although complicated by the appearance of apoptotic cells, would reveal diminished expression of larger numbers of haploid expressed genes.

It has been suggested that Trf2 and Taf7l cooperate to regulate spermatogenesis^37^. However, the complete arrest of spermatogenesis in *Trf2*^−/−^ mice contrasts with the progressive loss of reproductive potential of the *Taf7l*^−/y^ males^37^,^51^. Moreover, when we compared gene expression in the two lines only 15 genes were commonly down-regulated. Thus, in agreement with the very different phenotypes of these mice, Trf2 and Taf7l regulate distinct gene expression programs.

Increased expression of several general transcription factors may contribute to generally higher levels of gene expression in haploid cells^52^. Furthermore, several paralogs of general transcription factors exemplified by Trf2, Taf7l, and ALF are also specifically expressed in these cells. To better understand the function of Trf2, we propose to consider it as a testis-specific subunit of TFIIA and ALF. Trf2 has high affinity for TFIIA/ALF^23^ and immunopurification from testis extracts shows that it is a tightly associated integral subunit of TFIIA and ALF (ref. 35 and this study). Similarly, Teichmann also reported that when HeLa cells were engineered to stably express TRF2, it co-purified under stringent conditions with TFIIA^26^. Considering Trf2 as a testis-specific TFIIA/ALF subunit is also consistent with its observed recruitment to the PIC at haploid expressed genes. While some previous studies have considered Trf2 as specifically recruited to testis-specific genes (see for example^49^), our data rather support a model where testis-specific Trf2-containing TFIIA and/or ALF act as general factors that together with Tbp and other components of the PIC, promote optimal expression of haploid expressed spermatogenesis genes.

## Methods.

### Generation of *Trf2* ^tag/tag^ mice

A modified pL451vector containing a 6XHisTev3XFlag TAP-TAG followed by a neomycin resistance cassette flanked with two FRT sites was used to construct a targeting vector by sub cloning 5′ and 3′ arms of the *Trf2* locus. The 5′ arm was a 4 kb fragment of ending with the last amino acid of Trf2. The 3′ arm was a 5,7 kb fragment starting with the 3′ UTR of *Trf2*. Homologous recombination, ES cell screening and mouse generation were performed by standard procedures. Once mice with the germ-line mutation were identified they were crossed with mice ubiquitously expressing FLP recombinase to delete the neomycin resistance cassette. Mice were bred on a 129/SvPas background. All experimental procedures were approved by the French national ethics committee of the Ministère de l’Enseignement Supérieur et de la Recherche (CNEA). All experimental procedures involving mice were performed in accordance with institutional guidelines regarding the care and use of laboratory animals and with National Animal Care Guidelines (European Commission directive 86/609/CEE; French decree no. 87-848).

### Antibodies

The following antibodies were used in this study. For ChIP: Flag- M2 resin (Sigma), Trf2: in house rabbit polyclonal antibody 1727 raised against a peptide corresponding to Trf2 amino acids 115–136, gift from L. Tora, TBP: ab28175, Abcam and ab51841, Abcam, PolII: sc 9001X, Santa Cruz, H3K4Me3: 04-745, Millipore. For immunoblots the following antibodies were used. Trf2; 1727 as described above, TFIIA: rabbit polyclonal J17, gift from H.G. Stunnenberg, ALF: in house mouse monoclonal 2B11 as described^35^, Taf7l: in house mouse monoclonal 46TA as described^36^, TBP: ab51841 Abcam, TAF4: in house mouse monoclonal 32TA as described^53^. TAF1: ABE42, Millipore, Actb in house mouse monoclonal 1ACT-2D7, H3: ab1791 Abcam.

### Preparation of nuclear and cytoplasmic extracts

20 mouse testes were homogenized by douncing with a loose pestle in 10 mL hypotonic buffer (50 mM Tris pH8, 1 mM EDTA, 10 mM KCL, 1 mM DTT and 1X protease inhibitors (#11 873 580 001, Roche). After 10 min incubation on ice the suspension was centrifuged for 10 min at 4000 rpm and the supernatant kept as cytoplasmic extract. The nuclear pellet was washed once with hypotonic buffer and dounced in 2 mL high salt buffer (50 mM Tris pH8, 0,5 M NaCl, 0,5 mM EDTA, 20% glycerol, 1 mM DTT and 1X protease inhibitors) with a tight pestle. The suspension was incubated at 4 °C with agitation for 30 minutes and centrifuged at 13000 rpm for 5 min.

### Gel filtration

500 μl of cytoplasmic extracts containing 5–10 mg of protein were injected onto a equilibrated Superose 6 (10/300) column and run at 0.4 ml/min. 500 ul fractions were collected and analysed by western blot.

### Tandem immunoaffinity purification and mass-spectrometry

The fractions containing Trf2 and TFIIA/ALF from the gel filtration were pooled and subjected to tandem Flag-His purification. Tagged Trf2 was immunoprecipitated with anti-Flag M2-agarose (Sigma), eluted with Flag peptide (0.5 mg/mL), and further affinity purified with Talon superflow affinity resin (Clontech) and elution with 0.1 M EDTA. Purified complexes from the *Trf2*^tag/tag^ and control *Trf2*^+/+^ fractions were resolved by SDS-PAGE and stained using the Silver Quest kit (Invitrogen). Sections of gel from the lowest to highest molecular mass were systematically excised and analysed by mass-spectrometry either at the Taplin Biological Mass Spectrometry Facility (Harvard Medical School, Boston, MA) or the IGBMC mass-spectrometry facility.

### Chromatin preparation

Chromatin from mouse testes was prepared either by sonication or by digestion with MNase. Mouse testes were decapsulated in PBS and seminiferous tubules were put into a dounce containing 0.4% PFA in 1X PBS (for sonicated chromatin) or 1% PFA in 1X PBS (for MNase digestion) and homogenised with a loose pestle. Cross-linking was stopped with glycine (0.4 M final concentration). The cell pellet was collected by centrifugation for 5 min at 4000 rpm, lysed in 5 volumes of lysis buffer (50 mM Tris pH8, 10 mM EDTA, 1% SDS, protease inhibitors) and sonicated for 10 min on a Covaris sonicator. MNase digested chromatin was prepared by resuspending the cell pellet in 7 mL hypotonic buffer (50 mM Tris pH8, 1 mM EDTA, 10 mM KCL, 1 mM DTT) and douncing with tight pestle. The suspension was incubated for 10 min on ice and centrifuged for 5 min at 4000 rpm. Nuclei were washed once with 1X PBS and spin and resuspended in 2 volumes of MNase lysis buffer (50 mM Tris pH8, 0.5% Na deoxycholate). The lysate was sonicated for 30 sec as described above, CaCl2 and MgCl2 were added to 5 mM final concentration each and digestion was performed with MNase (10 gel units/uL final) (M0247S, New England BioLabs) for 10 min of incubation. The reaction was stopped by addition of EDTA to 10 mM final concentration. SDS (0.5% final) and protease inhibitors were added and suspension left on ice for 10 min and the centrifuged for 5 min at 13000 rpm. The supernatant was used for MNase digested chromatin.

### ChIP-seq

ChIP experiments were performed on sonicated or MNase digested chromatin as previously described. ChIP-reChIP was performed for Trf2 and Tbp according to the same protocol with minor modifications as follows. After first IP [anti Flag M2 resin (Sigma) or anti-TBP (ab28175, Sigma)] chromatin was eluted with a 3-fold smaller volume of elution buffer (1% SDS, 0.1 M NaHCO_3_). For Trf2, the eluate was diluted 10 times with ChIP-dilution buffer (1.1%Triton X100, 1.2 mM EDTA, 16.7 mM Tris-Hcl-pH8.0, 167 mM NaCl) and re-ChIPed with the polyclonal anti-Trf2 antibody 1727. For Tbp the second ChIP was performed using or anti-TBP (ab51841, Abcam). ChIP-seq libraries were prepared and sequenced on an Illumina Hi-seq2500 as single-end 50-base reads. After sequencing, peak detection was performed using the MACS software^54^ (http://liulab.dfci.harvard.edu/MACS/). Global clustering, meta-analyses and quantitative comparisons were performed using seqMINER and R (http://www.r-project.org/). The Taf7l ChIP-seq data is from GSE50807^37^.

### RNA-seq

Total RNA from mouse testes was isolated using the TriReagent (Molecular research center TR118). Libraries of template molecules suitable for high throughput DNA sequencing were created using “TruSeq™ Stranded Total RNA LT library prep kit with Ribo-Zero Gold” according to manufacturer’s user guide (Illumina Inc.). The libraries were loaded in the flow cell at 7pM concentration and clusters were generated in the Cbot and sequenced on the Illumina Hiseq2500 as single-end 50 base reads. Sequence reads mapped to reference genome mm9/NCBI37 using Tophat^55^. Data normalization and quantification of gene expression was performed using the DESeq 2 Bioconductor package^56^. Three independent biological replicates were analysed for each mouse genotype. Gene set enrichment analyses were performed using the BROAD javaGSEA standalone version (http://www.broadinstitute.org/gsea/downloads.jsp) and the curated C3 transcription factor target gene set collection (BROAD molecular signature database, MSigDbv4.0, http://www.broadinstitute.org/gsea/msigdb/index.jsp). For GSEA of *Trf2*^+/+^ versus *Trf2*^−/−^, we used the mean of the log2 fold changes of the biological replicates as metric for the H Hallmark gene sets of the BROAD javaGSEA tool with 1,000 permutations and the canonical pathway (cp) subcollection of the C2 curated BROAD molecular signature gene-set collection.

## Additional Information

**How to cite this article**: Martianov, I. *et al*. TRF2 is recruited to the pre-initiation complex as a testis-specific subunit of TFIIA/ALF to promote haploid cell gene expression. *Sci. Rep.* **6**, 32069; doi: 10.1038/srep32069 (2016).

## Supplementary Material

Supplementary Information

Supplementary Dataset
