# Supplementary material for: TRF2 is recruited to the pre-initiation complex as a testis-specific subunit of TFIIA/ALF to promote haploid cell gene expression
Source: Sci Rep. 2016 Aug 31;6:32069. doi: 10.1038/srep32069 (PMC5006001; doi:10.1038/srep32069)
Supplement: Supplementary Information [file srep32069-s1.pdf]

TRF2 is recruited to the pre-initiation complex as a testis-specific subunit of TFIIA/ ALF to promote haploid cell gene expression.

Igor Martianov[1], Amandine Velt[1], Guillaume Davidson[1], Mohamed-Amin Choukrallah[2], and Irwin Davidson[1]#*.

1. Department of Functional Genomics and Cancer, Institut de Génétique et de Biologie Moléculaire et Cellulaire. CNRS/INSERM/UDS. 1 Rue Laurent Fries, 67404 Illkirch Cédex. France.

2. The Friedrich Miescher Institute, Maulbeerstrasse 66, 4058 Basel, Switzerland.

Supplementary Figure 1. Specificity of tandem Trf2 ChIP. **A.** Flag-ChIP-qPCR at the *Rpl37* and *Znf512b* loci and a control intragenic locus using chromatin from *Trf2*$^{tag/tag}$ or *Trf2*$^{-/-}$ testis as indicated. **B.** The Flag-ChIPped material was re-chipped using Trf2 antibody. **C**. UCSC screen shot of ChIP-seq for Trf2, Pol II and Taf7l at the *Rpl37* and *Znf512b* loci. Sites that were bound by Trf2 in *Trf2*$^{tag/tag}$ testis chromatin and lost in *Trf2*$^{-/-}$ chromain are indicated by arrows.

A

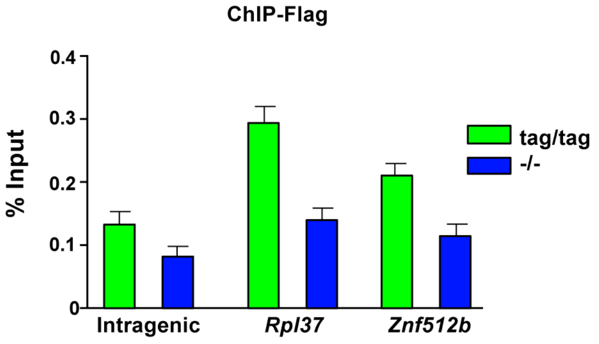
ChIP-Flag
% Input
0.4
0.3
0.2
0.1
0
Intragenic
Rpl37
Znf512b
tag/tag
-/-

B

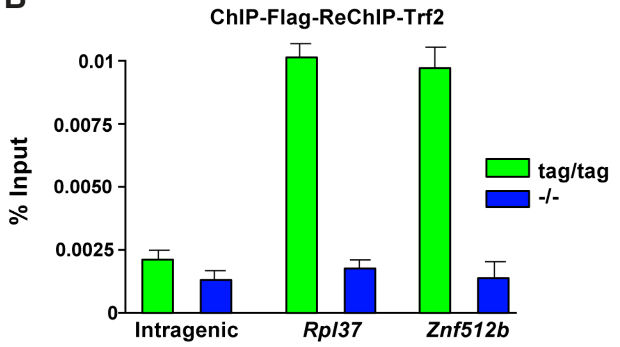
ChIP-Flag-ReChIP-Trf2
% Input
0.01
0.0075
0.0050
0.0025
0
Intragenic
Rpl37
Znf512b
tag/tag
-/-

B

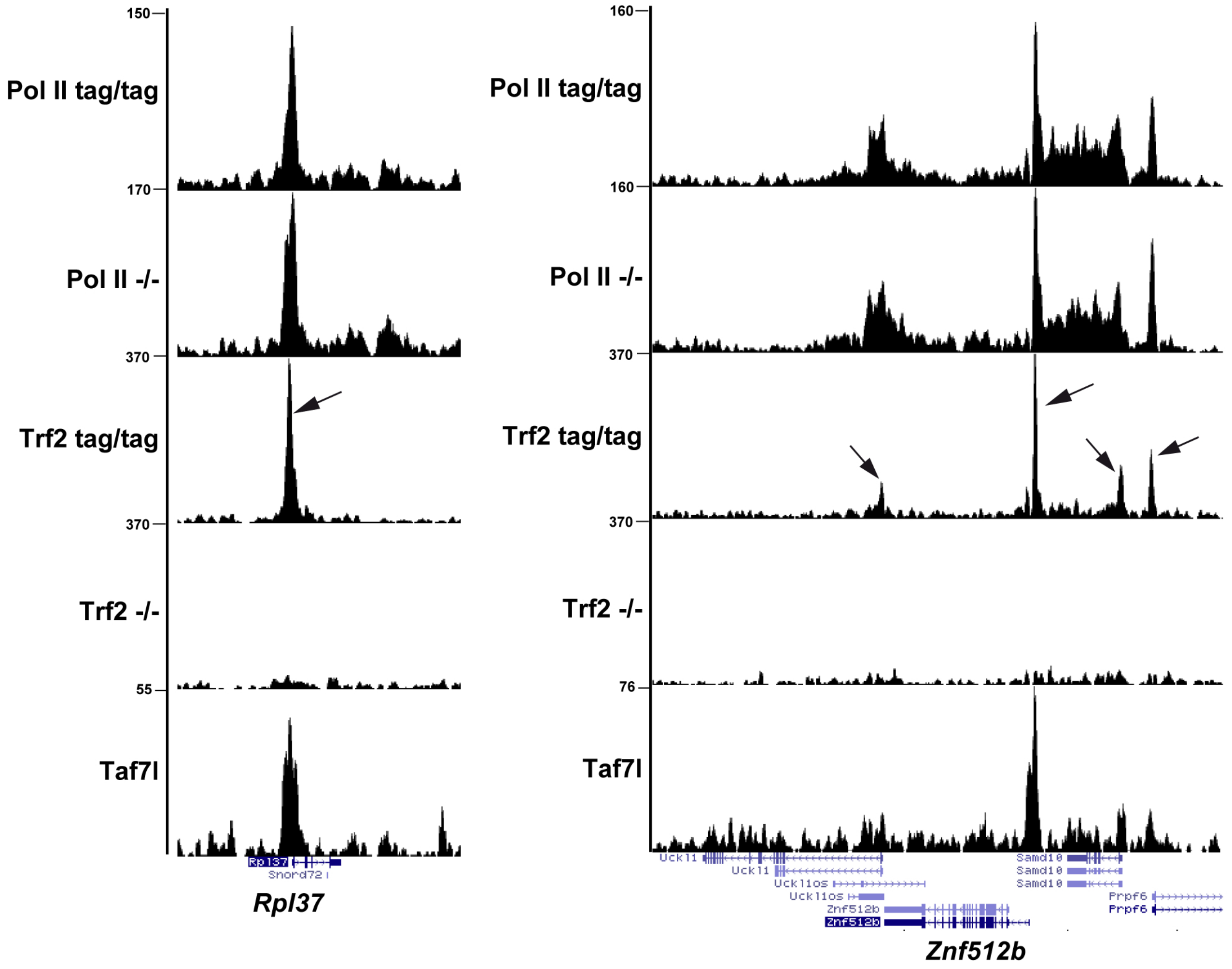
Pol II tag/tag
Pol II -/-
Trf2 tag/tag
Trf2 -/-
Taf7l
150
170
370
370
55
160
160
370
370
76
Rpl37
Snord72
Rpl37
Uckl1
Uckl1
Uckl1os
Uckl1os
Znf512b
Znf512b
Samd10
Samd10
Samd10
Prpf6
Prpf6
Znf512b
